# Social media language of healthcare super-utilizers

**DOI:** 10.1038/s41746-021-00419-2

**Published:** 2021-03-25

**Authors:** Sharath Chandra Guntuku, Elissa V. Klinger, Haley J. McCalpin, Lyle H. Ungar, David A. Asch, Raina M. Merchant

**Affiliations:** 1 Penn Medicine Center for Digital Health, Philadelphia, PA USA; 2 Department of Computer and Information Science, University of Pennsylvania, Philadelphia, PA USA; 3 Perelman School of Medicine, University of Pennsylvania, Philadelphia, PA USA; 4 Penn Medicine Center for Health Care Innovation, Philadelphia, PA USA; 5 The Wharton School, University of Pennsylvania, Philadelphia, PA USA; 6 Cpl Michael J Crescenz VA Medical Center, Philadelphia, PA USA

**Keywords:** Health care, Communication

## Abstract

An understanding of healthcare super-utilizers’ online behaviors could better identify experiences to inform interventions. In this retrospective case-control study, we analyzed patients’ social media posts to better understand their day-to-day behaviors and emotions expressed online. Patients included those receiving care in an urban academic emergency department who consented to share access to their historical Facebook posts and electronic health records. Super-utilizers were defined as patients with more than six visits to the Emergency Department (ED) in a year. We compared posts by super-utilizers with a matched group using propensity scoring based on age, gender and Charlson comorbidity index. Super-utilizers were more likely to post about confusion and negativity (D = .65, 95% CI-[.38, .95]), self-reflection (D = .63 [.35, .91]), avoidance (D = .62 [.34, .90]), swearing (D = .52 [.24, .79]), sleep (D = .60 [.32, .88]), seeking help and attention (D = .61 [.33, .89]), psychosomatic symptoms, (D = .49 [.22, .77]), self-agency (D = .56 [.29, .85]), anger (D = .51, [.24, .79]), stress (D = .46, [.19, .73]), and lonely expressions (D = .44, [.17, .71]). Insights from this study can potentially supplement offline community care services with online social support interventions considering the high engagement of super-utilizers on social media.

## Introduction

Super-utilizers are patients with frequent acute (i.e., emergency department) healthcare encounters, often because of complex physical, behavioral, and social needs. Parameters of defining healthcare super-utilizers vary across the literature, but the Center for Medicare and Medicaid Services (CMS) defines super-utilizers as “patients who accumulate large numbers of emergency department (ED) visits and hospital admissions, which might have been prevented by relatively inexpensive early interventions and primary care”.^1^ Super-utilization can result from or contribute to uncoordinated care and avoidable utilization of inpatient and emergency room services, and poorer health outcomes overall^2^. The cost implications of super-utilization are significant, with a 2012–2013 analysis^3^ in the United States (US) demonstrating that 50% of healthcare expenditures were attributed to just 5% of the population. Further costs are incurred with the common comorbidities seen among super-utilizers, including mental health and substance use diagnoses^4^.

Conventional approaches to managing this population rely on comprehensive care coordination, community-based care, and greater attention to complex social needs. However, combined approaches are difficult to implement and expensive^4^ because they are applied broadly, in contrast to narrower interventions targeting specific patient populations and their socio-contextual and behavioral needs. One example of this comprehensive approach that received national attention was carried out by the Camden Coalition of Healthcare Providers. Results of their randomized controlled trial revealed that the intervention—which involved care coordination among nurses, social workers, and community health workers—did not have any significant effect on reducing readmission rates 180 days following hospital discharge^5^. Patient navigator interventions targeting super-utilizers have shown mixed or moderate success in reducing hospital utilization^6,7^.

The Camden Coalition and others rely on the incorporation of social determinants of health into their proposed interventions^8^. However, as Iovan et al. (2019) found in a comprehensive review of super-utilizer interventions, most interventions target the downstream determinants surrounding patients’ material conditions (e.g. access to housing, food, and transportation), with only a select few offering interventions targeting the more fundamental social determinants of health with referrals to education, job opportunities, and vocational training^9–11^. Tackling these fundamental determinants requires a more targeted approach that can be tailored to the individual and engages the patient, their families, and caregivers beyond conventional healthcare visits.

Digital engagement is increasingly sought to effectively ‘hover’ over patients outside of their traditional healthcare encounters^12^. Smartphones, wearable devices, and social media may offer opportunities to engage patients, including super-utilizers, in care that meets them where they are—both physically and metaphorically—while simultaneously offering a more cost-effective and proactive approach to solving for patient needs. Facebook—used by 69% of adults in the US, of whom 74% report using it at least once a day^13^—provides both metadata and user-generated content, and thus offers a unique window into the lives of patients, and may reveal potential opportunities for interventions.

In this study, we sought to understand the online activity of consenting healthcare super-utilizers, specifically their day-to-day lifestyle, behaviors, and emotions by comparing their entire timeline of Facebook posts (quantified by open-vocabulary topics, dictionary-based psycholinguistic categories, linguistic markers of anger, stress, and loneliness expressions) with those of a matched group using propensity scoring based on Charlson comorbidity index, gender, and age.

## Results

### Characteristics of study subjects

We defined super-utilizers a priori as any patient who had six or more ED encounters within a 12-month time period within our urban health system^14^. Out of 1830 who shared Facebook data and electronic health records as part of the Social Mediome study cohort^15^, 109 participants met our criteria and were thus categorized as super-utilizers (median age of 28 and 83% women) (Table 1). The control group included 109 participants (median age of 31 and 86% women). In our cohort, super-utilizers had more documented diagnoses of injury and poisoning, respiratory symptoms, skin disorders, anxiety, depression, and documented drug use when compared to the control group (Chi-squared statistic significant at *p* < .001), consistent with previous findings on the prevalence of comorbidities among super-utilizers^1^. Super-utilizers also had on average two times more posts (*N* = 1537 posts/user) in their social media profile compared to the control group (*N* = 963 posts/user), significant at *p* < .05 two-tailed *t*-test.

**Table 1:** Demographic characteristics and diagnoses distribution of healthcare super-utilizers and the control group.

	Super-utilizers	Control
Total participants	109	109
Race		
African American	98 (89%)	83 (76%)
White	11 (11%)	23 (21%)
Other	0	3 (3%)
Females	90 (83%)	94 (86%)
Age range (yrs) and IQR	[20, 58]; 8	[20, 84]; 34
Age median (yrs)	28	31
Charlson Comorbidity Score range and IQR	[−16, 57]; 7	[−9, 28]; 5
Distribution of top diagnoses		
Injury and poisoning	92 (84%)	60 (55%)
Respiratory symptoms	81 (74%)	51 (46%)
Skin disorders	74 (67%)	45 (41%)
Anxiety	38 (34%)	15 (13%)
Depression	43 (39%)	23 (21%)
Drug abuse	21 (19%)	6 (5%)

A participant could have multiple documented diagnoses at each visit. Distribution of top diagnoses are significantly different across groups at *p* < 0.001.

### Differentially expressed language features in the super-utilizer group

#### Dictionary-based

Super-utilizers used more self-references, first person pronouns (Cohen’s D = .75, [0.47, 1.05]), words indicating present focus (D = .57, [.29, .86]), and function words such as adverbs (D = .53, [.26, .81]) and negations (D = .49, [.22, .78]) (Table 2). They also used words indicative of cognitive processes including differentiation (D = .47, [.2, .76]), tentativeness (D = .38, [.11, .66]), and discrepancies (D = .37, [.1, .64]).

**Table 2:** Linguistic Enquiry Word Count (LIWC) categories significantly associated with language used in Facebook posts of healthcare super-utilizers.

Category	Cohen’s D	95% CI
Pronouns		
1st person pronouns	0.75	[0.47, 1.05]
Impersonal pronouns	0.41	[0.14, 0.68]
Time orientation		
Present focus	0.57	[0.29, 0.86]
Perceptual processes		
Feeling	0.56	[0.29, 0.85]
Bodily processes		
Body	0.54	[0.26, 0.82]
Sexual	0.49	[0.22, 0.77]
Cognitive processes		
Differentiation	0.47	[0.20, 0.76]
Tentativeness	0.38	[0.11, 0.66]
Discrepancies	0.37	[0.1, 0.64]
Drives		
Reward	0.47	[0.20, 0.75]
Function words		
Adverbs	0.53	[0.26, 0.81]
Negations	0.49	[0.22, 0.78]
Negative emotions		
Swearing	0.35	[0.08, 0.63]

Only significant categories after Benjamini–Hochberg p-correction and *p* < 0.05 are shown.

#### Open-vocabulary

Compared with the control group, super-utilizers were more likely to post about confusion and negativity (‘erked’, ‘pissed’, ‘upset’, ‘confused’, ‘rite’, D = .65, 95% CI-[.38, .95]), self-reflection (‘mind’, ‘thinking’, ‘alot’, ‘much’, ‘head’, D = .63 [.35, .91]), avoidance (‘wanna’, ‘away’, ‘far’, ‘stay’, ‘cry’, D = .62 [.34, .90]) and swearing, D = .52 [.24, .79], sleep (‘fall’, ‘sleep’, ‘asleep’, ‘bed’, ‘down’, D = .60 [.32, .88]), seeking help and attention (‘need’, ‘help’, ‘someone’, ‘please’, ‘come’, ‘save’, D = .61 [.33, .89]), psychosomatic symptoms (‘pain’, ‘hurt’, ‘killing’, ‘ugh’, ‘feeling’, D = .49 [.22, .77]), and self-agency (‘make’, ‘sure’, ‘things’, ‘move’, ‘decisions’, D = .56 [.29, .85]) (Table 3). Some of the highly correlated words are colloquial variations used on social media (e.g., ‘erkerd’ and ‘rite’).

**Table 3:** Topics (clusters of co-occurring words) significantly associated with language used in Facebook posts of healthcare super-utilizers.

Topic Theme	Highly correlated words in topic	Cohen’s D (95% CI)	Example posts
Confusion and negativity	‘erked’, ‘pissed’, ‘upset’, ‘confused’, ‘annoyed’, ‘right’, ‘rite’, ‘now’	0.65 (0.38, 0.95)	*“Soo erked right now!”*
			*“I’m pissed right now”*
			*“Is cryin rite now”*
Self-reflection	‘mind’, ‘thinking’, ‘alot, ‘much, ‘head’, ‘what’	0.63 (0.35, 0.91)	*“Alot on my mind”*
			*“Cant sleep, mind racing”*
			*“Wishful thinking”*
	‘need’, ‘some’, ‘new’, ‘asap’, ‘something’, ‘drink’, ‘serious’	0.50 (0.23, 0.78)	*“I need a drink….. Damn oxycodone”.*
			*“I need a new hobby”*
			*“Need new friends asap”*
Avoidance	‘wanna’, ‘away’, ‘far’, ‘stay’, ‘cry’, ‘walk’, ‘scream’	0.62 (0.34, 0.90)	*“#phoneOff Don’t wanna be bothered”*
			*“Just want 2 scream”*
			*“Ugggg”*
Seeking help and attention	‘need’, ‘help’, ‘someone’, ‘please’, ‘come’, ‘save’	0.61 (0.33, 0.89)	*“boredddd ugh somebody save me”*
			*“Lord help me”*
			*“Somebody Call Me”*
	‘want’, ‘give’, ‘ask’, ‘whatever’, ‘deserve’, ‘chance’, ‘attention’	0.57 (0.30, 0.86)	*“i dont want you but. i cant let you go!”*
			*“Want something to eat”*
Sleep	‘fall’, ‘sleep’, ‘asleep’, ‘bed’, ‘down’, ‘watch’, ‘lay’, ‘tv’	0.60 (0.32, 0.88)	*“Goodnight fb Watchin TV Till I Fall Asleep.”*
			*“Going bac tosleep”*
	‘take’, ‘nap’, ‘care’, ‘woke’, ‘break’, ‘shower’, ‘sleepy’	0.51 (0.24, 0.79)	*“Finally nap time!”*
			*“Taking a nap tired”*
			*“ttyl fb nap time”*
Self-agency and decision-making	‘make’, ‘sure’, ‘things’, ‘move’, ‘decisions’, ‘trying’	0.56 (0.29, 0.85)	*“Need to make better decisions….. seriously”*
			*“Time to make some changes”.*
			*“DECISIONS DECISIONS NOW WHAT IM GOIN TO DO”*
			*“Check out my new business card”*
	‘own’, ‘business’, ‘care’, ‘ppl’, ‘stop’, ‘worry’, ‘handle’	0.48 (0.21, 0.76)	*“Handling business like an adult should…”*
			*“Time 2 stop bullshitting an handle my buisness”*
Swearing	‘f**k’, ‘s**t’, ‘nobody’, ‘give’, ‘everybody’, ‘point’	0.52 (0.24, 0.79)	“F**k Everybody Nobody Talk To Me”
			“F**k it #shruggs”
	‘s**t’, ‘people’, f**king’, ‘dumb’, ‘stop’, ‘stupid’	0.44 (0.17, 0.72)	*“I cant stop sleeping….always tired #ridiculous”*
			*“Ppl is so petty”*
Psychosomatic symptoms	‘pain’, ‘hurt’, ‘killing’, ‘stomach’, ‘ugh’, ‘feeling’, ‘pissed’	0.49 (0.22, 0.77)	*“My stomach hurts:(“*
			*“Ouch ouch ouchhhhhh”.*
			*“Tummy And Head Hurt,:’(“*
	‘better’, ‘feel’, ‘little’, ‘hope’, ‘makes’, ‘gets’, ‘worse’	0.57 (0.30, 0.85)	*“Feeling neglected”*
			*“feeling real crappy”*
			*“I feel unappreciated….”*

Effect size is measured using Cohen’s D. Topics were categorized into themes based on a review of the posts most associated with the topic. All categories shown are significant after Benjamini–Hochberg p-correction (*p* < 0.01).

#### Mental well-being

Super-utilizers were more likely to have posts containing language associated with anger (D = .51, [.24, .79]), stress (D = .46, [.19, .73]), and lonely expressions (D = .44, [.17, .71]). Language related to depression (D = .23, [.03, 0.5]) and anxiety (D = .20, [.06, .47]) was only slightly elevated compared to the control group.

## Discussion

In this study, we identified themes and contexts associated with ED super-utilizer posts on Facebook that reflected stress, anger, avoidance, attention-seeking, self-reflection, and health symptoms. Many of the topics reflect social-contextual challenges that may be contributing to healthcare seeking behaviors. Prior work has shown that super-utilizers are more likely to have complex physical, behavioral, and social needs^16^. Our work demonstrates that these complex circumstances are in fact reflected in the social media behaviors of this patient population as measured through linguistic characteristics that demonstrate stress, conflict, and loneliness. Future studies could investigate the extent to which social media posting and behavior—including language, images, and ‘lurking’ time—accurately reflect the lived experience of patients. Any approach involving personalized interventions would require significant technical infrastructure and thorough ethical review to guard against further stigmatization of an already vulnerable population.

Super-utilizers tend to have more severe and uncontrolled chronic illness^2^; the volume of language about psychosomatic symptoms posted by super-utilizers compared with the control group in this sample supports this finding. Attention-seeking language may reflect unmet needs in the daily experiences of super-utilizers and could also be a marker of loneliness, social isolation, or underlying mental health diagnoses. The burden of mental health in populations of super-utilizers has been well documented^2^, so the relationship between psychiatric conditions, social vulnerability, and language on social media is plausible.

### Implications on intervention design

Much of the existing literature uses payer data to identify commonalities among super-utilizer patient profiles^4,14,17^. Among the published interventions, these data are augmented with patient interviews and assessments to gauge access to resources (e.g., social supports, living and working circumstances, and food security), which can then inform a case management method for providing targeted support to the patient^14,18–20^. While our findings support the characterizations of super-utilizers published in previous literature, they also suggest a potential application in future targeted interventions. Utilizing nontraditional digital sources to characterize the expressions of super-utilizers may allow care teams—particularly social workers and care coordinators—to understand essential elements of a patient’s daily life that may allow for a more tailored course of action to address healthcare and other needs. Such a model would require patients to share social media data with their care teams, which has several technical and ethical ramifications.

Social media analysis can potentially be used to supplement offline community care services with online social support interventions considering the high engagement of super-utilizers on social media. Engaging patients online also holds potential for increased interactive support^21^. While exploring digital social support groups for cancer patients, online environments were found to provide a platform for asking questions, communicating personal experiences, and sharing emotions^22^. In harnessing the dynamic nature of these platforms, interventions targeted to super-utilizers could respond and adapt to these highly engaged patients in an easily accessible and familiar environment. Opportunities are also growing in the development of new digital health technologies. Prior work explored super-utilizer receptivity to digital technologies for care management and outlined key takeaways from focus groups including widespread interest in digital health tools, healthcare delivery navigation challenges, and age-based digital literacy^23^. Our data provide further insight into super-utilizers’ digital presence that could benefit future development of digital health technologies targeted to this population.

### Ethics and privacy

Maintaining privacy and confidentiality are critical when looking toward healthcare applications of social media data^24^. Potential stigmatization of already vulnerable service users once they have been flagged as potential super-utilizers could be problematic and should be guarded against. Specific guidelines for social media health research should include strict protocols around protection measures for sensitive data and deidentification whenever possible, as well as data storage on HIPAA-compliant servers^25^. Such safeguards are one approach for protecting against any downstream insurance or employment consequences in the event of data breaches. Furthermore, any personalized interventions utilizing such data should place high value on maintaining patient agency and avoid any prescriptive measures based unilaterally on social media insights. Lastly, it is important to preserve trust in the relationship between provider and patient, especially among vulnerable populations. A note of caution is that introducing social media data into the patient-physician relationship can result in a patient’s privacy feeling violated or influence a provider in their treatment^26^.

### Limitations

This study has several limitations. First, although the demographics of our sample are similar to the overall population served by the ED in urban hospitals^15^, our sample is not representative of the general population and is skewed towards younger African American females. Payer data revealed that super-utilizers with Medicaid coverage were older than other Medicaid patients, with an average age of 32.3 years for super-utilizers compared to 24.2 for patients with less than 6 hospital visits per year^14^. We prioritized matching on gender, the Charlson comorbidity score, and age (in that order). We found age and race to be significantly different across groups. In prior work, it was found that gender has the highest effect on language, but does not change a lot after 45 years, which was the reason for our characterization^27^. Previous literature found that super-utilizers, compared to ‘low-utilizers’, are more likely to be male and African American^28^ and Hispanic/Latino^29^.

Second, the EHR data for visits is obtained from one health system whereas patients might have received care from other systems not captured in our analysis. Third, though the exclusion of non-English speaking participants avoids cultural confounders considering the specific recruitment location of participants, it introduces sampling bias. Further, patients who are willing to share social media data may tend to be “over-sharers” so that the conclusions drawn may not be generalizable to all ED super-utilizers, and especially because eligibility was limited to English-speakers and English language posts.

In summary, social media language offers a window into patients’ characteristics that cannot be gleaned from their health records alone and may eventually lead to new ways to identify needs at the individual or population level. Healthcare super-utilizers’ social media posts reveal themes that suggest lifestyles, behaviors, and emotions that reflect negativity, conflict, sleep deprivation, and psychosomatic symptoms. While these findings need to be replicated in other studies before implementing interventions, this study is a step towards considering the inclusion of patient-generated data, with explicit consent, in understanding healthcare needs and sequelae—providing insight and a comprehensive view of the challenges these patients face beyond their medical presentation.

## Methods

### Study design and setting

The study was approved by the University of Pennsylvania Institutional Review Board. Using a convenience sample framework, from March 2014 through December 2017 patients receiving care in the emergency department (ED) of an urban academic hospital system were approached about participating in a study to merge social media and Electronic Health Records (EHR) data^15^. All participants gave their written informed consent to use their data for this study.

### Selection of participants

We retrieved Facebook status updates up to 5 years prior to the ED index visit for all participants who consented to share their Facebook posts (*N* = 4587). We did not access data from the Facebook pages of study participants’ friends or from posts on the study participants’ pages made by anyone other than the participant. We excluded non-English posts and selected users with a minimum of 400 words, determined from prior work to be the minimum threshold for reliably predicting user traits from language^30^, retaining 1830 participants with Facebook data.

Extracting data from the EHR, we identified the ED visits for these participants which coincided with years when they also had Facebook data. We first identified all years (from 2009 to 2016) in which participants had six or more ED visits. For each patient, we obtained primary ICD-9 codes of every ED and inpatient visit available in the EHR. Then, we used these ICD-9 codes to obtain the diagnoses by mapping them onto the categories in Elixhauser comorbidity codes^31^. We used these categories to identify differences in diagnoses across super-utilizer and control groups. Further, using the same ICD-9 codes, we calculated the Charlson comorbidity index to obtain a measure of severity of disease for every patient. We characterized patients with six or more ED visits in any year from 2009 to 2016 as super-utilizers—as most of them had contiguous hospital visits in these years^16^. Since healthcare utilization varies based on demographics and severity of illness, we identified a propensity score matched group of control users based on the Charlson comorbidity index, gender, and age of our super-utilizer set in a retrospective case-control manner.

### Linguistic attributes

We characterized posts using three sets of language features: (a) dictionary-based psycholinguistic features, (b) open-vocabulary topics^32^, and (c) mental well-being attributes, such as anger, anxiety, depression, stress, and lonely expressions by applying previously developed predictive models^33–35^.

#### Dictionary-based

From each post, we extracted the relative frequency of words/tokens. We removed words used by less than 1% of users. We then compared the posts of the super-utilizer and control groups against the 73 psycholinguistic categories from the Linguistic Inquiry Word Count (LIWC)^36^. For each, we measured the proportion of tokens (including words, emoticons etc.) represented in each LIWC category.

#### Open-vocabulary

We also used an open-vocabulary approach. Two hundred latent Dirichlet allocation (LDA) topics (groups of co-occurring words) were generated using Facebook posts contributed by patients from a prior study^32^. The LDA generative model assumes that posts contain a combination of topics, and that topics are a distribution of words. Since the words in a post are known, topics, which are latent variables, can be estimated through Gibbs sampling. We use the Mallet implementation of the LDA algorithm, adjusting one parameter (alpha = 5) to favor fewer topics per post. All other parameters were kept at their default. An example of such a model is the following set of words (‘tuesday’, ‘monday’, ‘wednesday’,…) which clusters together days of the week by exploiting their similar distributional properties across tweets. We calculated the topic distribution of each user aggregated across all posts.

#### Mental well-being attributes

We used automatic text-regression methods developed in previous works to assign to each user scores on the depression^35^, anxiety^37^, anger, stress^34^, and lonely expressions^33^.

### Identifying differentially expressed language features in the super-utilizer group

Posts from the same years were used for both case and control groups—2009–2016. We designed this as a person-level analyses and each individual was counted only once: 109 cases and 109 controls. All language features were extracted and compared at the individual level. Each linguistic attribute and mental well-being attribute were used as input in a logistic regression model. The models were setup to predict super-utilizers (i.e., group was the dependent variable). In accordance with conventional linguistic analysis, we used a *p*-value of <.05 for LIWC and mental health attributes and *p* < .01 for topics, after adjusting for multiple comparisons using Benjamini–Hochberg correction, to identify potentially meaningful associations. We calculated Cohen’s D associated with the super-utilizer’s group with the control group as reference, for each retained attribute^38^.

### Reporting summary

Further information on research design is available in the Nature Research Reporting Summary linked to this article.

## Supplementary information

Reporting Summary

## Data Availability

Deidentified data necessary to reproduce the results contained in the document are available upon request. We will not, however, share individual-level Facebook data as it contains potentially identifying information about patients enrolled in the study.

## Code availability

Analysis code is released as part of the Differential Language Analysis Toolkit (http://dlatk.wwbp.org).
