# Supplementary material for: Social media language of healthcare super-utilizers
Source: NPJ Digit Med. 2021 Mar 25;4:55. doi: 10.1038/s41746-021-00419-2 (PMC7994843; doi:10.1038/s41746-021-00419-2)
Supplement: Supplementary file 1 — Reporting Summary [file 41746_2021_419_MOESM1_ESM.pdf]

nature research

Corresponding author(s): Sharath Chandra Guntuku

Last updated by author(s): Dec 7, 2020

Reporting Summary

Nature Research wishes to improve the reproducibility of the work that we publish. This form provides structure for consistency and transparency in reporting. For further information on Nature Research policies, see our Editorial Policies and the Editorial Policy Checklist.

Statistics

For all statistical analyses, confirm that the following items are present in the figure legend, table legend, main text, or Methods section.

n/a	Confirmed	
☐	☒	The exact sample size (n) for each experimental group/condition, given as a discrete number and unit of measurement
☐	☒	A statement on whether measurements were taken from distinct samples or whether the same sample was measured repeatedly
☐	☒	The statistical test(s) used AND whether they are one- or two-sided<br>*Only common tests should be described solely by name; describe more complex techniques in the Methods section.*
☐	☒	A description of all covariates tested
☐	☒	A description of any assumptions or corrections, such as tests of normality and adjustment for multiple comparisons
☐	☒	A full description of the statistical parameters including central tendency (e.g. means) or other basic estimates (e.g. regression coefficient) AND variation (e.g. standard deviation) or associated estimates of uncertainty (e.g. confidence intervals)
☐	☒	For null hypothesis testing, the test statistic (e.g. F, t, r) with confidence intervals, effect sizes, degrees of freedom and P value noted<br>*Give P values as exact values whenever suitable.*
☒	☐	For Bayesian analysis, information on the choice of priors and Markov chain Monte Carlo settings
☒	☐	For hierarchical and complex designs, identification of the appropriate level for tests and full reporting of outcomes
☐	☒	Estimates of effect sizes (e.g. Cohen's d, Pearson's r), indicating how they were calculated

Our web collection on statistics for biologists contains articles on many of the points above.

Software and code

Policy information about availability of computer code

Data collection	Data from consenting individuals was collected using one-click authorization of a Facebook application using their API: https://developers.facebook.com/docs/graph-api/
Data analysis	Language analysis code is released as part of the Differential Language Analysis Toolkit (http://dlatk.wwbp.org).

For manuscripts utilizing custom algorithms or software that are central to the research but not yet described in published literature, software must be made available to editors and reviewers. We strongly encourage code deposition in a community repository (e.g. GitHub). See the Nature Research guidelines for submitting code & software for further information.

Data

Policy information about availability of data

All manuscripts must include a data availability statement. This statement should provide the following information, where applicable:

- Accession codes, unique identifiers, or web links for publicly available datasets
- A list of figures that have associated raw data
- A description of any restrictions on data availability

De-identified data necessary to reproduce the results contained in the document are available upon request. We will not, however, share individual-level Facebook data as it contains potentially identifying information about patients enrolled in the study.

Field-specific reporting

Please select the one below that is the best fit for your research. If you are not sure, read the appropriate sections before making your selection.

☐ Life sciences ☒ Behavioural & social sciences ☐ Ecological, evolutionary & environmental sciences

For a reference copy of the document with all sections, see nature.com/documents/nr-reporting-summary-flat.pdf

Behavioural & social sciences study design

All studies must disclose on these points even when the disclosure is negative.

Study description	Quantitative: Retrospective Case-Control Study
Research sample	Patients receiving care in the emergency department (ED) of an urban academic hospital system.<br>N = 218<br>Age range: 20-84<br>% Female: 83<br><br>Sample is representative of the patient population served by the academic hospital system. For further details, see:<br>Padrez KA, Ungar L, Schwartz HA, et al. Linking social media and medical record data: a study of adults presenting to an academic, urban emergency department. BMJ Qual Saf. 2016;25(6):414-423.
Sampling strategy	Convenience sampling.
Data collection	Patients receiving care in the emergency department (ED) of an urban academic hospital system were approached about participating in a study to merge social media and Electronic Health Records (EHR) data. All participants gave their written informed consent to use their data for this study.
Timing	March 2014 through December 2017
Data exclusions	Users with less than 400 words in their Facebook timeline were excluded. 400 words was determined from prior work to be the minimum threshold for reliably predicting user traits from language.
Non-participation	No participants dropped out of the study sample used in this paper.
Randomization	Since healthcare utilization varies based on age, gender, and severity of illness, we identified a propensity score matched group of control users based on Charlson comorbidity index, gender, and age of our super-utilizer set in a retrospective case-control manner.

Reporting for specific materials, systems and methods

We require information from authors about some types of materials, experimental systems and methods used in many studies. Here, indicate whether each material, system or method listed is relevant to your study. If you are not sure if a list item applies to your research, read the appropriate section before selecting a response.

Materials & experimental systems

n/a	Involved in the study	
☒	☐	Antibodies
☒	☐	Eukaryotic cell lines
☒	☐	Palaeontology and archaeology
☒	☐	Animals and other organisms
☐	☒	Human research participants
☒	☐	Clinical data
☒	☐	Dual use research of concern

Methods

n/a	Involved in the study	
☒	☐	ChIP-seq
☒	☐	Flow cytometry
☒	☐	MRI-based neuroimaging

Human research participants

Policy information about studies involving human research participants

Population characteristics	See above
Recruitment	Patients receiving care in the emergency department (ED) of an urban academic hospital system were approached about participating in a study to merge social media and Electronic Health Records (EHR) data. All participants gave their written informed consent to use their data for this study.

	Although the demographics of our sample are similar to the overall population served by the ED in our urban hospitals, our sample is not representative of the general population and is skewed towards younger African American females.
Ethics oversight	University of Pennsylvania

Note that full information on the approval of the study protocol must also be provided in the manuscript.
